# Medical Grievances and Suggested Remedies

**Published:** 1900-11-17

**Authors:** R. Brudenell Carter

**Affiliations:** Harley Street, W.


### Editor's Letter-Box.

[Our Correspondents are reminded that prolixity is a great bar to publication, and that brevity of style and conciseness of statement greatly facilitate early insertion.]

### MEDICAL GRIEVANCES AND SUGGESTED REMEDIES.

To the Editors of *The Hospital.*

Sirs,—I have lately made a humble endeavour, in compliance with a request from the Editors of the *International Journal of Ethics*, to describe the accepted standards of conduct in the medical profession, and to explain the sanctions by which those standards are upheld. On the whole my endeavour has not been unfavourably received; but it has called forth from the *Lancet* the criticism that I "have given no advice to the profession on the crucial point of what rule, if any, is to be made about men who hold club appointments which place them in the position of paid servants of laymen who demand from them work which they cannot do for remuneration which does not remunerate them." The *Lancet* agrees with me that the Medical Council "cannot possibly treat members of the profession who choose to undersell their professional services as persons guilty of infamous conduct in a professional sense;" but it cannot acquiesce in the view (which I have never put forward, and which the *Lancet* does not ascribe to me) "that nothing need be done." You have yourselves taken a somewhat similar ground, and have plainly indicated that, in your judgment, medical practitioners should be restrained (as I understand, by some form of enactment) from undertaking, for clubs or other employers, more work than they can properly accomplish.

It did not fall within the scope of my article in the *International Journal* to consider questions of this class but it is none the less true that they are eminently worthy of consideration. The practices in question border very closely upon dishonesty, and they tend to bring the medical profession, as a whole, into disrepute.

It is, nevertheless, to me inconceivable that they can be restrained by any form of legal process. It would be a wholesome exercise for those who think this possible to endeavour to frame the clauses of an Act of Parliament which would effect the purpose in view, and would do so without contravening accepted principles of natural justice or of public policy. No such clauses have ever yet been suggested, even by the most ignorant of the vulgar itinerant spouters who seek a profitable notoriety by bawling for coercion at the meetings of obscure medical societies; and it is manifestly useless to discuss coercion in the abstract until we are in possession of some definite suggestions as to the methods by which it could be effected. A law that no medical man should undertake more work than he could properly accomplish would have no other operation than to provide an unrivalled field for the ingenuities of legal advocacy and of judicial interpretation. It is obvious either that the offence would defy definition, or that any definition would include large classes of cases to which the law could not reasonably be intended to apply. The ordinary contract of a club doctor is that he will attend the members of the club when they are ill; and the underlying assumption is that the average number ill at any one time will not be in excess of his capabilities. If the locality were visited by an epidemic, and the average amount of sickness were greatly exceeded, what would be his duty then? Would it be to make the best fight he could with difficulties, and to work without sparing himself until further help could be obtained, or should he leave a portion of the sick untended? If the latter were his proper course, how should those who were to be untended be selected? Suppose there were a clause in his contract that a qualified assistant should be provided for him as soon as the sick members of the club exceeded a certain number; what would be his duty if the contingency occurred, and the assistant were not forthcoming? Such questions as these present themselves during even the first glance at the difficulties of the subject; and the answer given to them by the public, as well as by every medical practitioner worthy of belonging to the profession, would be that, at all hazards, duty to the sick must be discharged as far as the limits of human endurance would allow. In the noble words which I have formerly quoted from Sir James Paget, the doctor must not "neglect his duties for the sake of ease." It must be remembered, moreover, that such questions would not arise only in club practice or in badly remunerated practice, but quite as conceivably in private practice of the highest class, when claims of an unusual character are made upon those by whom it is conducted. No one would have anything but praise for the private practitioner who, on the occurrence of an epidemic in his locality, devoted himself unstintingly to his work, or would have anything but censure for him who, in such circumstances, refused to visit a fresh patient in a family that he was accustomed to attend, on the score that he was tired and needed rest. In the case of war, again, what would be thought of the doctor who turned his back upon the twentieth wounded man for no better reason than that he had attended to nineteen already? It is easy to say that no prohibitory law would be intended to apply to such conditions; but how would it be possible to devise a form of words which would render it inapplicable to them, or which could not be wrested into such applicability by personal spite or animosity?

A difficulty of equal magnitude would stand in the way of any attempt to define the amount of work which an individual doctor might reasonably and properly undertake. The capacities of different men are infinitely various. A man who has had a good night's rest is fit to accomplish much more than he would be after sitting up all night with a midwifery case. A man of good health, sound frame, clear head, and methodical habits will easily get through twice as much as one in whom these conditions are not fulfilled. The deliberate man cannot compete with the quick man, the muddle-headed with the clear-headed, the potterer with the alert. On such grounds as these, which might be almost indefinitely multiplied, it seems to me to be impossible that the law should ever act in restraint of the freedom of a practitioner to undertake as much as he pleases, and to undertake it for such payment as choice or necessity may induce him to accept. I cannot see how any restraining statute could be framed which would not be a curtailment of the proper liberties of Englishmen, and, as I stated above, in contravention alike of natural justice and of public policy. When the form of such a statute is laid before the profession by any *soi-disant* reformer, it will become possible to examine and to criticise its provisions. Until that time comes discussion of the subject must be unfruitful, and, even then, it would be very doubtful to what extent Parliament would be disposed to assent to the desires of amateur medical legislators.

Dismissing for the present, therefore, as outside the domain of practicability, any hope of legislative interference with the internal affairs of the profession, and remembering that the Medical Council would unquestionably be prevented by the Courts from exercising its highly penal jurisdiction in restraint of ordinary freedom, we may not unnaturally turn to the medical corporations and licensing bodies, and inquire to what extent they possess the power, and are likely to possess the will, to control the actions of their graduates or licentiates. A Bill for strengthening their hands has been prepared by the Medical Council, and is understood not to be unfavourably regarded by the Privy Council, but it has not yet become law. The Royal Colleges of Physicians and Surgeons, in all the divisions of the United Kingdom, being able to lay down conditions of membership or licentiateship, possess considerable powers, and are not averse to exercise them in proper cases; but the powers of the Universities are very limited, and are vested in bodies not essentially medical, and not necessarily in sympathy with medical aspirations. The Society of Apothecaries has no disciplinary powers at all, and forms, therefore, the weakest link in the chain of corporations. It is not unreasonable to suppose that any straining of their authority, on the part of the Royal Colleges, would tend to throw the less ambitious order of practitioners almost wholly into the arms of the Society, whose licentiates cannot be required to subscribe to by-laws or conditions, or to recognise any other authority over their conduct than that of the law of the land. The practical incapacities of the corporations have suggested to some ardent persons the desirableness, on grounds alleged to be "ethical," of superseding all other qualifications by a "State Examination," and they have endeavoured to persuade themselves or others that the State would subject the holders of its licences to conditions which the corporations are unable or unwilling to enforce. The reply is that the State, by which I mean the Imperial Parliament, has never shown the smallest inclination to undertake the task of controlling the members of any profession, but has invariably left them to control themselves, with such assistance, in the way of organisation, as charters were able to afford. Strictly speaking, indeed, the license of the Society of Apothecaries may be regarded as a State qualification, the power and duty of conferring it having been given by Act of Parliament; while it is the only qualification in existence which all respectable persons are entitled to obtain, without any other condition than that of satisfying the examiners, and to retain without any other condition than that of avoiding a conviction for felony or misdemeanour, or the equally penal jurisdiction of the General Medical Council.

I have endeavoured to assign reasons which convince me that no penal or coercive powers exist, and that no penal or coercive legislation can be expected, by which the abuses incidental to excessive competition in medical practice are likely to be diminished. If I may ask for space on another occasion I will endeavour to show in what directions we may legitimately look for improvements which we all admit to be required by the opinion of the profession, and which, if they could be effected, would undoubtedly improve its position in the estimation of the public.—Your most obedient servant, R. Brudenell Carter.

Harley Street, W., October 1900.

